# Tackling Waste of Fruits and Vegetables in Chilean Households with Tailored Informational–Enablement Interventions: A Randomized Controlled Pilot Study

**DOI:** 10.3390/foods15152733

**Published:** 2026-08-04

**Authors:** Carolina Fredes, María Isidora Benavente, Fernanda Rudolph, Gabriela Méndez, Loreto Rojas, María Ignacia Pérez, Daniela Avello, Rodrigo Fernández-Verdejo, Francisca Echeverría

**Affiliations:** 1 Departamento de Nutrición y Dietética, Escuela de Ciencias de la Salud, Facultad de Medicina, Pontificia Universidad Católica de Chile, Santiago 7820436, Chile; cpfredes@uc.cl (C.F.); ibenaventea@uc.cl (M.I.B.); fernanda.rudolph@uc.cl (F.R.); gmndez@uc.cl (G.M.); lorojass@uc.cl (L.R.); mcperez7@uc.cl (M.I.P.); rfernandev@uc.cl (R.F.-V.); 2 Departamento de Terapia Ocupacional, Escuela de Ciencias de la Salud, Facultad de Medicina, Pontificia Universidad Católica de Chile, Santiago 7820436, Chile; daniela.avello@uc.cl; 3 Pennington Biomedical Research Center, Louisiana State University, Baton Rouge, LA 70808, USA

**Keywords:** consumption, food waste, tailored intervention, behavioral interventions

## Abstract

Randomized Controlled Trials (RCTs) and direct measurement of waste are the gold standards for evaluating food waste interventions. This pilot RCT explored the effects of a 3-week tailored informational–enablement intervention to reduce the causes and amount of fruit and vegetable waste in households in Santiago, Chile. Households were randomized to intervention (*n* = 23) or control (*n* = 23). Instrumental Activities of Daily Living, fruit and vegetable consumption, and perceived causes of waste were assessed by questionnaire. Avoidable waste was quantified by direct weighing at baseline, post-intervention, and observational follow-up. At baseline, median (25th–75th percentiles) avoidable waste was 153 (128–180) (g/day per person for fruits and 102 (95–124) for vegetables. Purchase was the most frequently reported cause of waste. At post-intervention, the change in vegetable waste (Δwaste) was lower in the intervention vs. the control group (−6 (−9, −3) vs. 3 (−5, 10) g/day per person; *p* = 0.0129), without difference for fruits (*p* = 0.0895). Observational follow-up showed decreases in avoidable fruit and vegetable waste from post-intervention to month 6 (*p* < 0.001) and to month 9 (*p* < 0.001). In conclusion, the tailored informational–enablement intervention reduced avoidable vegetable waste in the studied Chilean households. Follow-up measurements could contribute to capturing longer-term changes in fruit and vegetable waste.

## 1. Introduction

Food waste represents a global challenge with substantial economic, environmental, and social implications [1]. Globally, approximately one-third of food produced is lost or wasted, equivalent to 1.3 billion tons each year [2]. Food loss refers to reductions in edible food during production, post-harvest handling and storage, and processing. In contrast, food waste occurs at the retail and final consumption stages (households and food service), when food that is fit for human consumption is discarded [1]. Food loss and waste (FLW) reflect an inefficient use of natural resources and account for nearly 50% of global greenhouse gas emissions from food systems, thereby contributing to climate change [3,4]. Moreover, FLW persists in a world where ~828 million people are undernourished, and ~676 million live with obesity, underscoring a critical disconnect between food production, food security, and responsible consumption [5,6].

FLW occurs throughout the food supply chain. Fruits and vegetables are the most wasted food groups worldwide due to their high perishability and limited post-harvest shelf life [2,7]. These losses are particularly concerning given their high nutritional value. Fruits and vegetables are essential sources of vitamins, minerals, dietary fiber, and bioactive compounds [8,9]. Daily consumption of at least 5 servings of fruits and vegetables helps prevent non-communicable diseases and reduces overall mortality, underscoring the importance of not wasting these food groups [10].

At the consumption level, households are responsible for a significant share of fruit and vegetable waste. Estimates vary by region; the avoidable waste of fruits and vegetables ranges from 30% of total household waste in Dutch households to 70% in Japan [11,12,13,14]. There are multiple causes of food waste in households. The main causes are a lack of purchase planning, excessive food preparation, and inadequate storage [15,16,17]. Households therefore face an urgent challenge in adopting individual behavioral changes towards food waste and implementing more sustainable food-handling practices on a daily basis (e.g., activities of daily living, such as purchasing and preparing meals).

Informational interventions are among the most widely used strategies to reduce household food waste, and include educational campaigns, meal-planning promotion, and guidance on proper food storage [18]. However, the effectiveness of informational interventions has been variable and often limited due to a lack of robust study designs (e.g., randomized controlled trials, RCTs), reliable and accurate measurements of food waste (e.g., direct weighing), and poor long-term follow-up [19,20,21]. Furthermore, evidence from RCTs suggests that informational interventions may be more effective when education is combined with other intervention components, such as enablement (i.e., providing tools and reducing barriers beyond education and training), which are more effective at reducing food waste in high-income countries [21].

A systematic review analyzed how interventions can influence consumer behavior regarding food waste, highlighting the importance of linking waste causes to consumer routines, identifying individual motivations to promote behavior change, and involving consumers in the co-design of interventions [22]. Because interventions targeting food consumption and food waste are more likely to be effective when they address specific drivers, tailoring has been proposed to align content and tools with individual routines and barriers [22,23,24]. In a prior study conducted in a fresh-food market, we observed that planning and storage were the main causes explaining surplus and waste. Therefore, the tailored intervention focused on the identified drivers by reducing the grocers’ initial stock of fruit and vegetables using tailored informational interventions [24]. Accordingly, tailoring can be operationalized by using specific determinants (e.g., purchasing, storage, and preparation) to prioritize and adapt intervention content and practical tools to each household’s routines and barriers. More broadly, research on household food-waste reduction should account for household characteristics, assess the drivers of waste, and implement household-tailored interventions. Tailored interventions provide information matched to the specific needs and context, thereby improving relevance and enhancing intervention effectiveness [23,24,25].

The limited evidence from Chile indicates that fruits and vegetables are the most wasted food groups at the consumption level [26], as reported in other countries [11,12,13,14,27,28]. In Chile, per capita domestic availability of fruits (203 g/day) and vegetables (201 g/day) is estimated to be adequate. Nevertheless, consumption remains low and fruit and vegetable waste is high [29]. A pilot study in 15 households in Chile showed that food waste (mean ± standard deviation) was 2.38 ± 4.60 kg/week per family, with fresh fruits and vegetables being the most frequently wasted (1.14 ± 1.84 kg/week) [27]. This evidence is particularly relevant considering that 85% of the Chilean population does not meet the recommendation of consuming at least five servings of fruits and vegetables per day, with a mean of one serving of fruits and two of vegetables per day [30]. Consequently, interventions aimed at reducing fruit and vegetable waste are important not only for sustainability but also for supporting adherence to the “5-a-day” recommendation and improving health outcomes. In this context, a recent pilot study (*n* = 33 households) evaluated the effects of different educational videos about fruit recipes in low-income households in Chile [31]. The videos increased fruit and vegetable purchases, reduced fruit waste, and increased vegetable waste, using self-reporting methods (i.e., purchase and waste records for one week). However, the study did not include a control group, direct measurement of waste, or follow-up beyond the immediate post-intervention period.

To address this gap, we conducted a pilot RCT study to explore the effects of a 3-week tailored informational–enablement intervention on the reported causes and directly measured amount of fruit and vegetable waste in households in Santiago, Chile. We designed interventions based on the causes of waste identified in each household to address the following question: What is the effect of a 3-week tailored informational–enablement intervention to prevent fruit and vegetable waste, compared to no intervention, on the causes and amount of waste in Chilean households? The preliminary hypothesis was that a tailored informational–enablement intervention, based on the causes of waste, would reduce the amount of fruit and vegetable waste in households in Santiago, Chile. Thus, this study aimed to explore the effects of a tailored informational–enablement intervention to reduce the causes and amount of fruit and vegetable waste in households in Santiago, Chile.

## 2. Materials and Methods

### 2.1. Design

**Figure 1:**
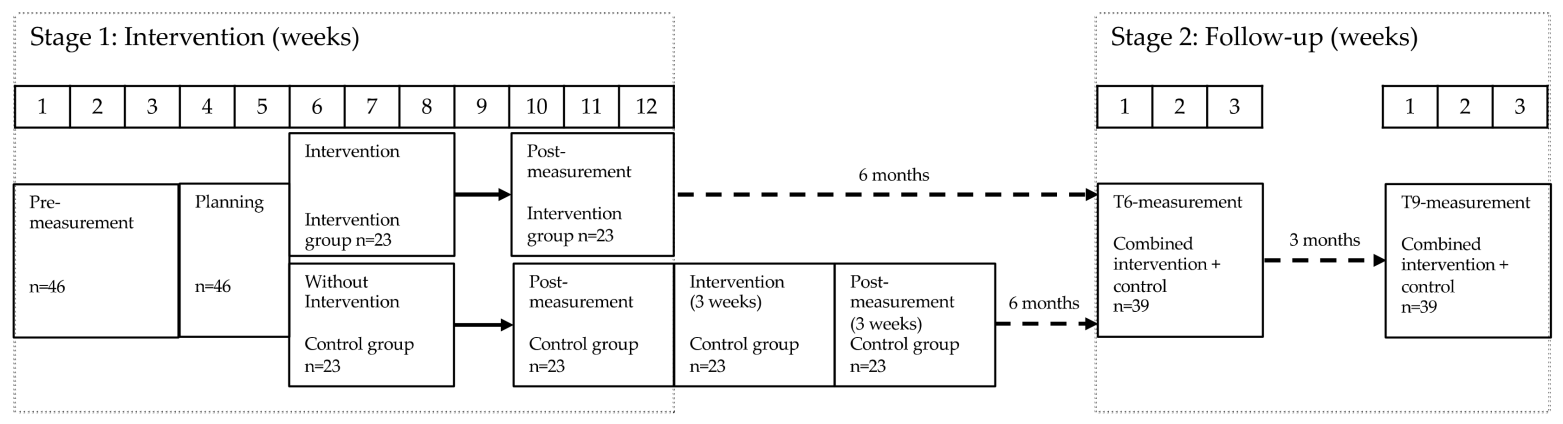
Study timeline and measurement points. Stage 1 comprised pre-intervention (baseline) measurement, planning, a 3-week tailored informational–enablement intervention (intervention group, *n* = 23) or no intervention (control group, *n* = 23), and post-intervention measurement (T0). Stage 2 included follow-up measurements at 6 months (T6) and 9 months (T9) after completion of the intervention; follow-up measurements were conducted in all households (intervention and control).

We conducted an experimental, randomized, controlled, repeated-measures pilot study [32]. Fruit and vegetable waste was first quantified in 46 households during a 3-week pre-intervention measurement period. Three questionnaires were administered at this time point: (a) Instrumental Activities of Daily Living (IADL), (b) fruit and vegetable consumption, and (c) causes of waste. Based on the results of these questionnaires, we planned tailored interventions to reduce household fruit and vegetable waste, in line with the recommendation of consuming at least 5 servings of fruits and vegetables per day [33]. The intervention-planning phase lasted 2 weeks. After enrollment and completion of the baseline assessment, households were assigned in a 1:1 ratio to either the tailored informational–enablement intervention for 3 weeks (*n* = 23; intervention group) or the waitlist-control group (*n* = 23; no intervention) using a simple manual randomization procedure. No blocking or stratification was used. Because of the nature of the intervention, participants and study personnel were not blinded to group allocation. One week after completion of the intervention, waste quantification and the questionnaire on the causes of waste were repeated in all households (i.e., post-intervention measurement). Then, the informational–enablement intervention was implemented for 3 weeks in the 23 households of the waitlist control group, and one week later, waste quantification was repeated in this group. We conducted observational follow-up measurement to quantify fruit and vegetable waste 6 and 9 months after each household completed the intervention. During the nine months of follow-up, the research team and undergraduate students in an FLW course reinforced the intervention recommendations through telephone calls and/or household visits. Figure 1 summarizes the study timeline.

### 2.2. Participants and Sample Size

Participants were households located in Santiago, the Metropolitan Region of Chile, and were recruited using convenience sampling. Inclusion criteria were households with: (1) two or more residents; (2) an adult household head aged 18–75 years; and (3) a household head with a basic proficiency in cellphone messaging. Exclusion criteria were households with: (1) a household head who was not functionally independent; and (2) residents who do not consume fruits and vegetables. We posted infographics on social media that illustrated the research procedure to invite households to participate. Recruitment occurred via cell phone messaging or calls. Each household head who agreed to participate signed an informed consent form approved by the Ethics Committee of the Pontificia Universidad Católica de Chile (research protocol ID 230125009).

For this pilot study, we initially calculated that a sample size of 59 households was required to detect problems arising with a 5% probability and a 95% confidence level, using a previously published equation [34]. Given this sample size, we calculated our sensitivity to detect differences between two independent groups (control vs. intervention) using G*Power version 3. Using the Mann–Whitney U test with 29 and 30 households per group, an α of 5% and a β of 20%, the effect size (between-group difference/standard deviation) was 0.76.

The recruitment allowed a sample of 48 households. Of them, 47 households completed the intervention and 46 the post-measurement. Thus, considering 46 households and a 95% confidence level, we calculated the probability of detecting problems [34]. The probability of detecting problems was 6%. Then, we used G*Power version 3 to calculate the sensitivity to detect differences between two independent groups (control vs. intervention). Using the Mann–Whitney U test with 23 households per group, α = 5%, and β = 20%, the effect size was 0.87. This represented a difference between households that was equivalent to 0.87 times the measurement’s variability. Consequently, if there were actual differences but of a smaller magnitude between groups (i.e., effect size lower than 0.87), we were underpowered to detect them.

### 2.3. Intervention

We designed an intervention to promote behavioral change towards fruit and vegetable waste. The intervention was mainly informational, assuming that providing recommendations for better purchase, preparation, consumption, and storage of fruits and vegetables could reduce avoidable household fruit and vegetable waste [35,36]. We combined informational content with enablement, which comprised practical tools intended to increase capability and opportunity to apply the recommended behaviors—thereby reducing barriers to implementation (e.g., household-specific purchase lists and actionable recipes). This approach was intended to facilitate purchase organization and the reuse of leftovers and edible peels in meals, helping to prevent the generation of fruit and vegetable waste. The context was households, the intervention lasted 3 weeks, and the total study duration (from the baseline measurement) was 49 weeks (May 2023 to April 2024; Figure 1).

**Figure 2:**
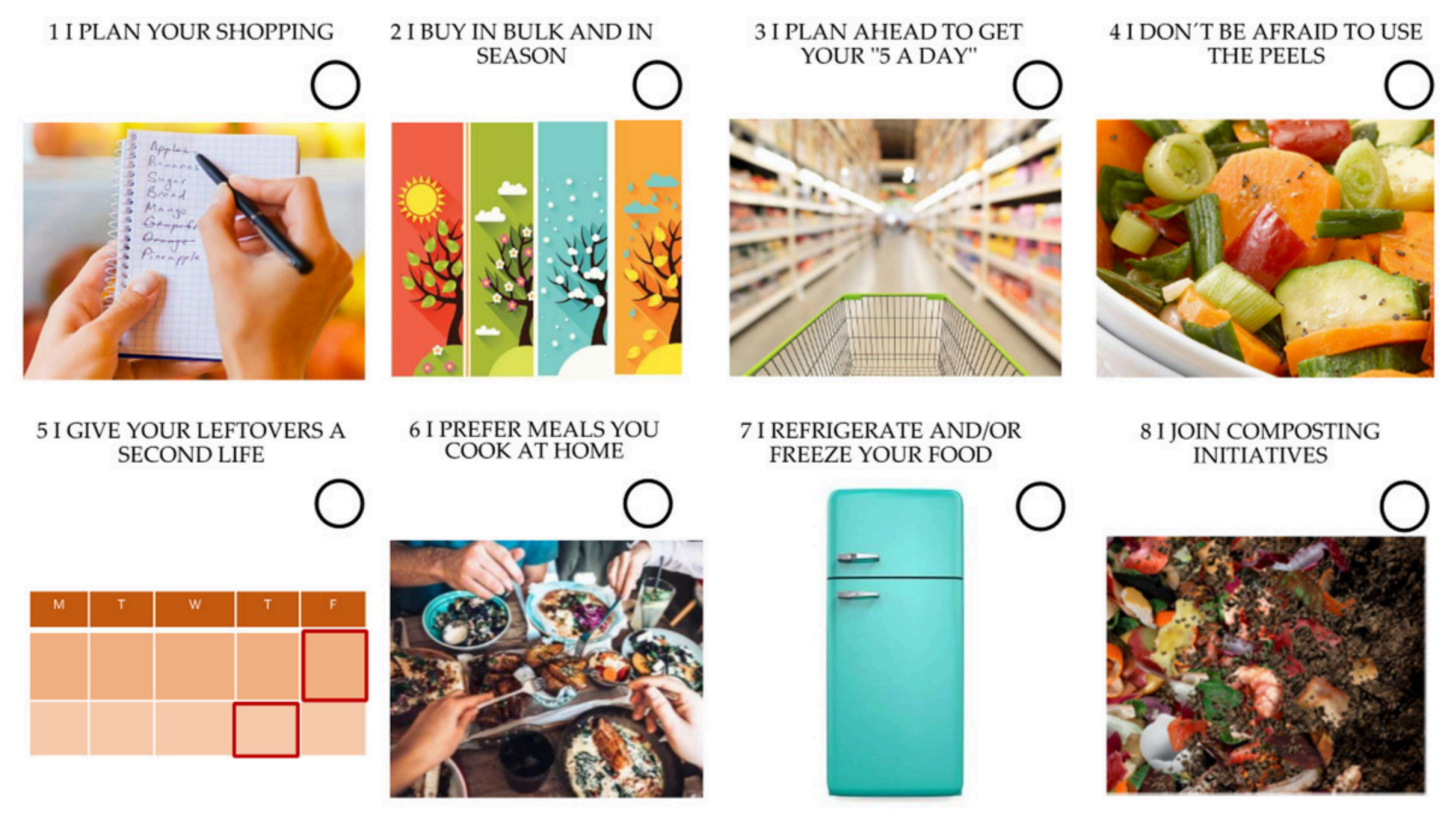
Summary of messages to prevent or reduce fruit and vegetable waste.

The four major causes of waste (purchase, preparation, consumption, storage) were considered as the intervention pillars. The information included recommendations to improve each pillar based on the existing literature [18,19,22,37,38,39,40,41,42,43]. These recommendations focused primarily on adopting habits to prevent or reduce avoidable waste, because this is the most environmentally preferable approach [44]. Moreover, recycling recommendations (e.g., vermicomposting) were considered for households that desired to complement habits for preventing or reducing avoidable waste with the management of unavoidable waste (e.g., inedible fruit and vegetable peels). In addition to informational content, the intervention included enablement tools to facilitate implementation. The enablement tools included a household-specific fruit and vegetable purchase list (accounting for local availability, purchase frequency, the five-a-day recommendation, and household size) and a set of recipes to encourage the use of leftovers and commonly discarded edible peels (e.g., tomato and potato peels). Tailoring was guided by each household’s responses to the causes-of-waste questionnaire (Section 2.5), prioritizing the pillar(s) with the highest driver burden. For example, households with frequent excess or unplanned purchasing received greater emphasis on the purchasing pillar. Intervention content and tools were delivered over 3 weeks through written materials (booklet and recipes; Figure 2) and oral activities (a meeting with the household head, an online educational activity, and a visit to a vermicompost center).

During the 3-week intervention period, the control group had no contact with the researchers.

### 2.4. Direct Quantification of Fruit and Vegetable Waste

**Figure 3:**
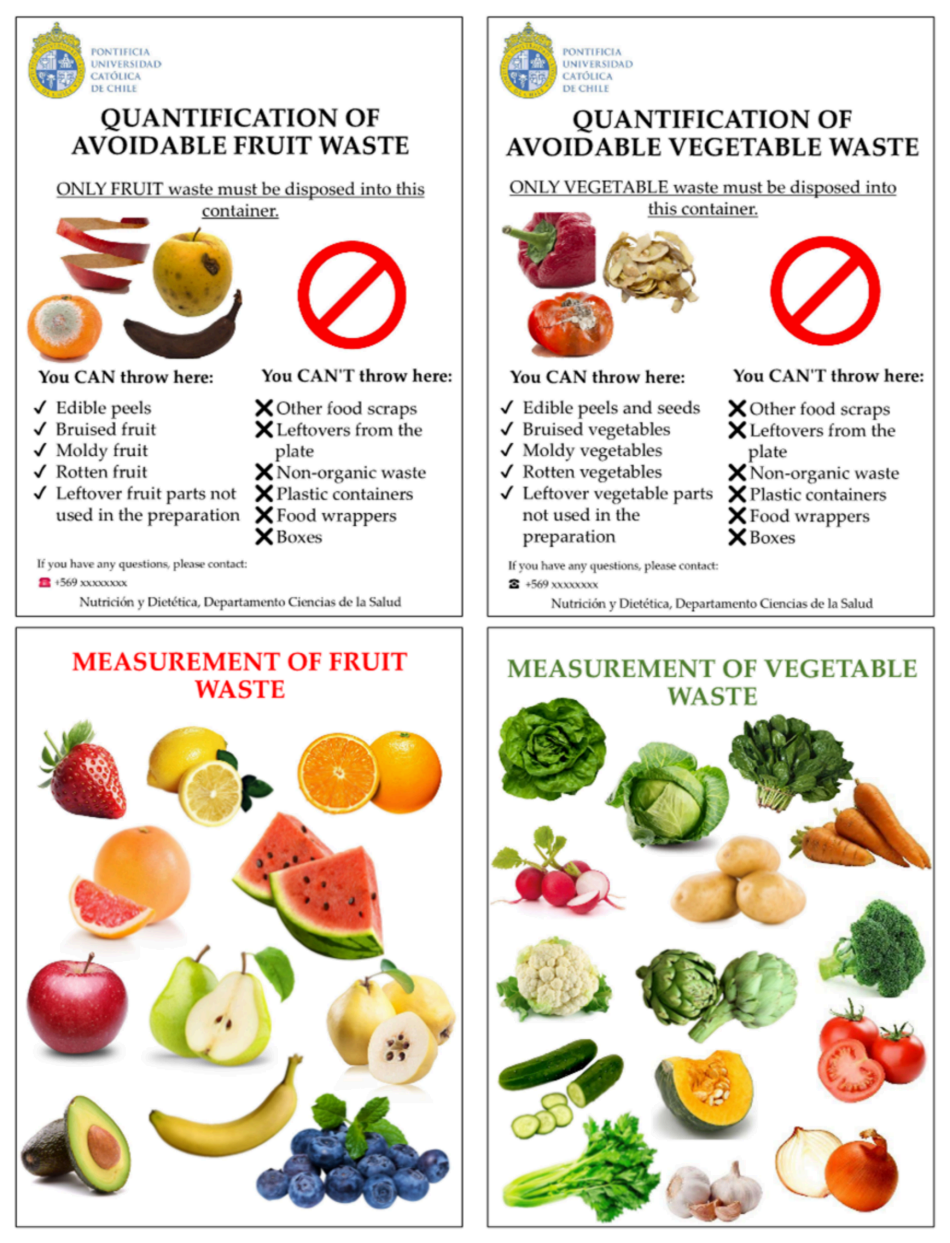
Infographics delivered to households to guide families on the management of fruit and vegetable waste.

To quantify waste, we used a weighing method, which is considered suitable for high-accuracy monitoring when researchers have direct access to solid food waste [45]. Households had two plastic bags available for collecting, including (a) avoidable fruit waste and (b) avoidable vegetable waste, over one week. Avoidable waste was defined as fruits or vegetables and their edible parts discarded during meal preparation or during cleaning of the refrigerator and pantry. The details of what should be deposited in each container were explained to each household head. Additionally, we provided infographics illustrating what could and could not be thrown away in each container, as well as which products corresponded to fruits or vegetables (Figure 3). Potatoes were included in the vegetable category due to their high Chilean household consumption frequency. Each household head could also contact the research staff to ask questions during the measurement week. To minimize desirability bias, all research procedures were explained to the household heads, emphasizing that food waste was highly probable to occur in their households and that we would measure it regardless of whether the amount appeared small or large.

During the measurement in each household, the containers were inspected and weighed on an electronic kitchen scale (Misato, SF400 model, Yongkang, China) on-site (days 3 and 7 of each measurement week). Based on the accumulated waste from days 3 and 7, avoidable waste was calculated for each household and divided by the number of residents (g/day per person). During each measurement, the household head provided information on their weekly purchases of fruits and vegetables. We performed the same procedure to quantify fruit and vegetable waste at the intervention and follow-up stages (Figure 1).

### 2.5. Questionnaires to Explore IADL, Fruit and Vegetable Consumption, and the Causes of Waste

The questionnaires were compiled into a form with five sections: (1) personal information, (2) IADL, (3) fruit and vegetable consumption, (4) perceived reasons that explain fruit and vegetable waste, and (5) open-ended comments. The form was administered face-to-face to each household head on the last day of waste measurement by the research team.

For IADL, we considered three questions about shopping (buy independently, enough financial resources to buy fruits, and enough financial resources to buy vegetables) and three questions related to cooking (prepare food independently, choose not to prepare food, and enjoy cooking activities) from the Lawton-Brody IADL Scale [46], modifying the 0 to 8 scale to yes/no responses. This questionnaire has been validated in Brazil, and a Spanish version was validated in Spain. However, it has not been validated in the Chilean population [47]. The fruit and vegetable consumption frequency questionnaire has been validated in the Chilean population and was part of the “Diet” section of the Chilean National Health Survey [30]. Each household head reported their own daily number of fruit and vegetable servings and consumption frequency, using graphical material to support their responses. From the data, we calculated the number of servings of fruit and vegetables per day and the frequency of consumption per week of the head of the household. The household head’s servings and consumption frequency of fruits and vegetables were considered representative of household consumption.

As for the causes of household fruit and vegetable waste, the questionnaire was adapted from published questionnaires, but has not been validated [24,48,49]. The questionnaire considered questions organized in four major causes of waste: (a) 3 questions about purchases (excess, incorrect, and unplanned purchase); (b) 3 questions about preparation (few ways to prepare, many inedible parts, and very difficult to prepare); (c) 5 questions about consumption (bad smell (e.g., rottenness), bad appearance, served many times a week, not consumed on time, and rarely consumed due to low acceptance); and (d) 4 questions about storage (more perishable compared to other foods, lack of refrigeration capacity, inadequate refrigeration due to equipment malfunction, and lack of order and visibility of food in the pantry or refrigerator). All questions were asked for each of three fruit categories (citrus, pomace, banana) and seven vegetable categories (leafy, fruits, stems, inflorescence, bulbs, roots/tubers, potato). For each question, participants were instructed to reply “yes” if they identified the cause, “no” if they did not, or “NA” if the item (e.g., banana) was not used in their household. For each major cause of waste (purchase, preparation, consumption, storage), we calculated an index for fruits and another for vegetables to reflect how relevant the cause was at each time point:

Index (%) = (Number of “yes”/Total number of valid questions) × 100%(1)

where Index (%) represents the index for each cause (purchase, preparation, consumption, storage) calculated separately for fruits and vegetables et each time point; Number of “yes” represents the number of questions answered with a “yes”; and Total number of valid questions represents the number of questions without an NA response. For example, for the major cause “purchase” among fruits, there was a total of 9 questions: 3 questions about purchase × 3 fruit categories. If the participant answered “yes” to 6 questions and “no” to 3 questions, the index would be: (6 “yes”/9 total) × 100% = 66%. Thus, the index ranges from 0% to 100%, where higher values suggest that the cause explained a greater proportion of the waste.

### 2.6. Data Analysis

Due to the small sample sizes, we used nonparametric tests [50]. We used GraphPad Prism version 10.5.0. A *p*-value < 0.05 was considered statistically significant.

Data on continuous variables are presented as medians (25th–75th percentiles). For each household, we calculated the change (Δ = post − pre) in each cause-specific index (%) and for the avoidable waste measurements (g/day per person). We used the Mann–Whitney U test to compare the ΔIndex and the ΔWaste between groups, separately for fruits and vegetables. These comparisons between groups allowed us to explore the effect of the intervention on the causes and the amount of waste.

Follow-up analyses were conducted to describe changes in avoidable waste over time after intervention exposure and to assess medium-term maintenance. We used the Friedman test to compare avoidable waste (g/day per person) across repeated measurements at post-intervention (T0) and follow-up at 6 months (T6) and 9 months (T9). When the Friedman test was significant, Dunn’s multiple-comparisons test was used for post hoc comparisons of T0 vs. T6 and T0 vs. T9.

## 3. Results

### 3.1. Households, IADL, and Consumption of Fruits and Vegetables

Household heads were mostly women (85%) with a median age of 48 years old. Most participants were born in Chile (98%), had a post-secondary educational level (89%); 72% were employed, and 91% had a monthly income higher than CLP 500,000 (USD 555). Seventy percent of participants reported good memory, but fewer than half in the intervention group did. Regarding the number of residents, household heads reported living in households of four (2–4) residents. Table 1 shows the characteristics of household heads.

The IADL indicated that over 80% of participants independently managed complex daily tasks such as purchasing and cooking, had the choice not to prepare food (i.e., may receive help to prepare food in the household or buy ready-to-eat food), and enjoyed cooking activities (Table 2). Note that fruits and vegetables were affordable for ~90% of participants. The frequency of consumption for fruits was 7 (4–7) days/week, and for vegetables was 7 (5–7) days/week. Fruit intake was 2 (1–2) servings/day, and for vegetables was 2 (1–2) servings/day, with a total fruit and vegetable consumption of 4 (3–5) servings/day. This resulted in nearly 30% of participants meeting the dietary recommendation of five fruits and vegetables per day.

### 3.2. Change in the Causes of Waste of Fruits and Vegetables

Table 3 shows the index of causes of waste before the intervention. Before the intervention, participants reported that purchasing was the main cause of waste for fruits and vegetables, associated with excess, incorrect, or unplanned purchases. Reasons associated with preparation, consumption, and storage were less reported by all participants. Therefore, the tailored intervention focused on improving the purchase pillar.

**Figure 4:**
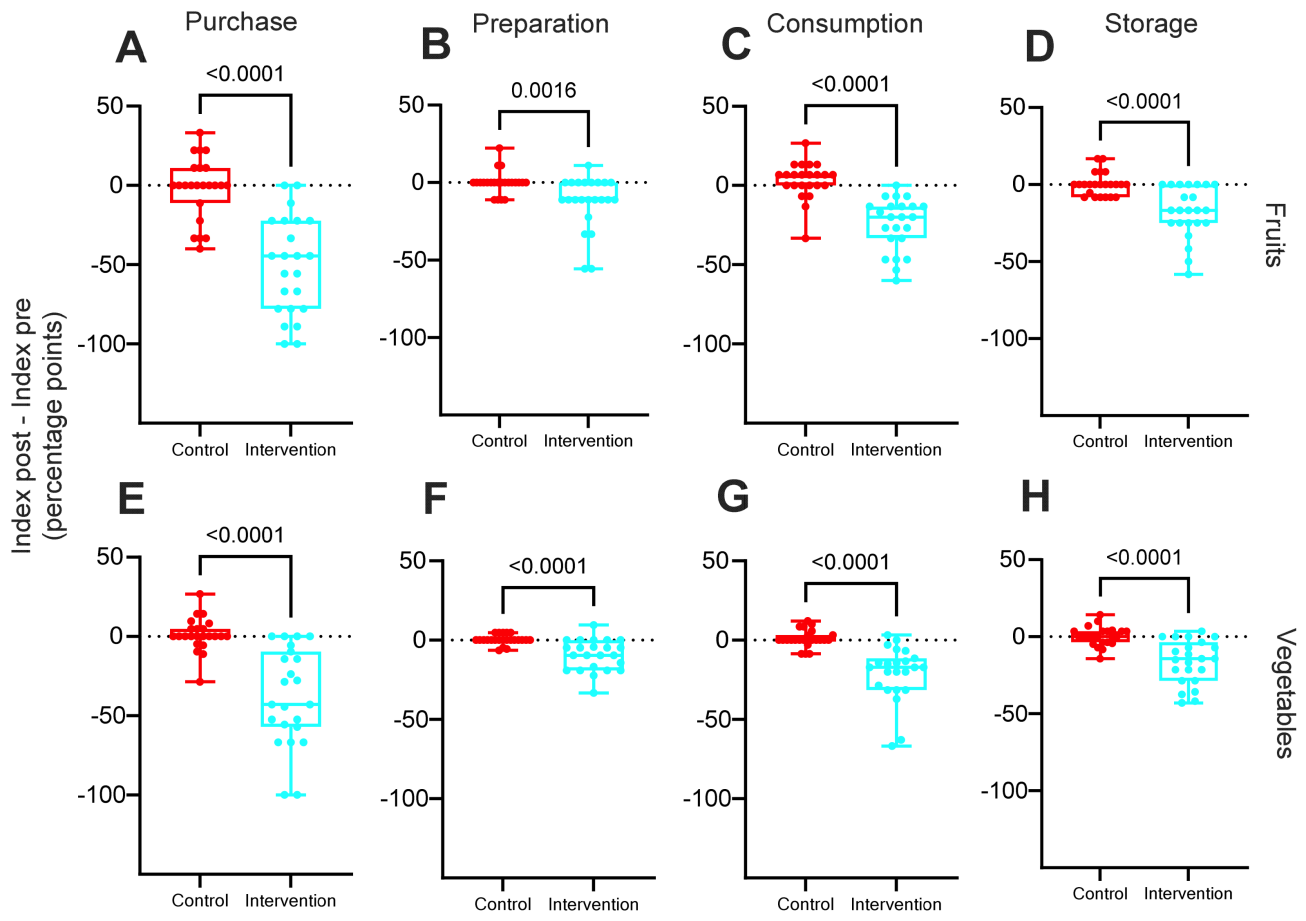
Change in the index (post–pre) for each of the causes of waste: purchase (**A**), preparation (**B**), consumption (**C**), and storage (**D**) for fruits and purchase (**E**), preparation (**F**), consumption (**G**), and storage (**H**) for vegetables. Red lines represent the control group and light blue lines represent the intervention group. Boxes represent the 25th and 75th percentiles, with a horizontal line denoting the median; whiskers denote the minimum and maximum values. Circles represent raw data points. Mann–Whitney U Test was used for comparison between groups, *p* < 0.05.

Following the intervention, the ΔIndex for each cause was lower in the intervention group compared with the control group (Figure 4). The major differences between the intervention group versus the control group were observed in fruit purchase (–44 percentage points; Figure 4A) and vegetable purchase (–43 percentage points; Figure 4E).

### 3.3. Change in Avoidable Fruit and Vegetable Waste

**Figure 5:**
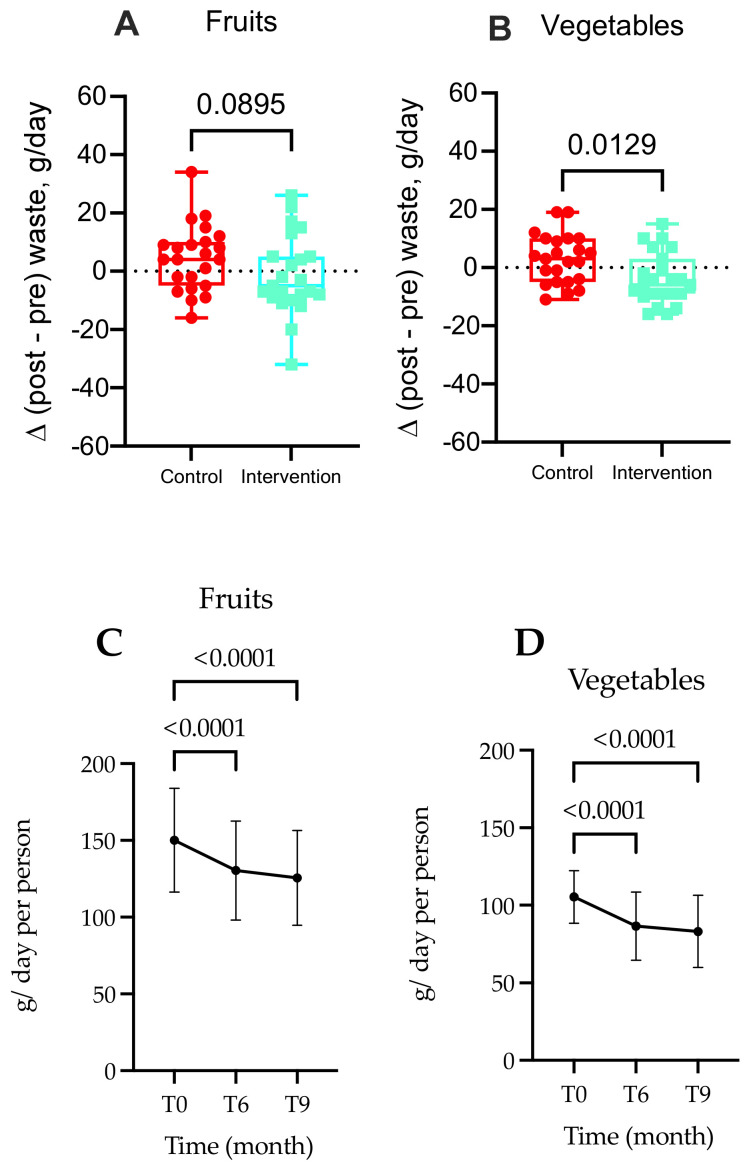
Change (Δ) in avoidable waste (post − pre) for fruits (**A**) and vegetables (**B**), and follow-up of avoidable waste (g/day per person) from post-intervention (T0) to 6 months (T6) and 9 months (T9) for fruits (**C**) and vegetables (**D**). Red lines represent the control group and light blue lines represent the intervention group. In panels (**A**) and (**B**), boxes represent the 25th and 75th percentiles; the horizontal line denotes the median; whiskers indicate the minimum and maximum values; and filled circles and squares represent individual data points for the control and intervention groups, respectively. In panels (**C**) and (**D**), points represent median values, and error bars represent the 25th–75th percentiles. The Mann–Whitney U test was used for between-group comparisons in panels (**A**) and (**B**). Dunn’s multiple-comparisons test was used for post hoc comparisons of T0 vs. T6 and T0 vs. T9 in panels (**C**) and (**D**).

Before the intervention, the avoidable waste was 153 (128–180) g/day per person for fruits and 102 (95–124) g/day per person for vegetables (Table 3). Following the intervention, the ΔWaste for fruits was not different between the intervention and the control group (–5 (–9, 5) and 4 (–5, 10) g/day per person, respectively; Figure 5A). The ΔWaste for vegetables, however, was lower in the intervention than in the control group (–6 (–9, 3) vs. 3 (–5, 10) g/day per person; Figure 5B). The effect size of between-group differences for fruit ΔWaste was 0.25, compared with 0.78 for vegetable ΔWaste. Notably, this difference in effect size is explained by both smaller between-group differences and larger variability in fruits than in vegetables (fruits: mean difference = 3.13 g/day, pooled standard deviation = 12.45 g/day; vegetables: mean difference = 6.74 g/day, pooled standard deviation = 8.60).

The follow-up stage showed a decrease in avoidable waste (g/day per person) for fruits from the post-intervention measurement (T0) to month 6 (T6) (*p* < 0.0001) and from post-intervention to month 9 (T9) (*p* < 0.0001) (Figure 5C). Similarly, there was a decrease in avoidable waste for vegetables from T0 to T6 (*p* < 0.0001) and T9 (*p* < 0.0001) (Figure 5D). This revealed that after 9 months post-intervention, the avoidable waste was 124 (106–148) g/day per person for fruits and 80 (63–97) g/day per person for vegetables. Thus, it could be estimated that the reduction in fruit and vegetable waste was 24 and 22 g/day per person, respectively, after 9 months of follow-up.

## 4. Discussion

Preventing or reducing avoidable waste is the most environmentally preferable approach for tackling food waste [44] and contributes to the Sustainable Development Goal 12, target 12.3 on responsible consumption and production aimed at halving FLW by 2030 [51]. This research provides evidence on the feasibility of tailored informational–enablement interventions for the design of larger studies and for potential scalability to reduce food waste.

In this pilot RCT study, we explored the feasibility of implementing a 3-week tailored informational–enablement intervention. We repeated direct-weighing measurements while generating preliminary estimates of intervention effects on avoidable fruit and vegetable waste in Santiago, Chile. We also considered an observational follow-up to describe post-intervention trajectories in waste levels over the medium term. Recruitment did not reach the initially projected number of households. With the available resources and the time allotted for project implementation, 48 households were recruited. In our case, disseminating the project through social media was insufficient to attract participants. Other strategies, such as the use of monetary and non-monetary incentives, could be evaluated for future research, as described in previous studies [31,52]. Gillies et al. [52] systematically reviewed strategies to improve participant retention in RCTs. They concluded that retention rates between 80% and 95% require particular attention, whereas retention below 80% may compromise trial validity. In the present study, 46 of the 48 recruited households completed the intervention stage (46/48 = 95.8%), indicating high short-term retention. However, 39 of 46 households remained in the study during the observational follow-up (39/46 = 84.8%), which falls within the range that warrants attention. Therefore, results should be interpreted with caution. These findings highlight the need to strengthen retention strategies in future household food-waste trials. Besides monetary and non-monetary incentives, a newspaper article about the trial and regular telephone contact with participants could be evaluated as strategies to support retention in larger studies [52].

Our findings suggest that a tailored informational–enablement intervention can reduce the frequency of the causes of fruit and vegetable waste and the amount of avoidable vegetable waste in households. Note that the follow-up showed a decrease in avoidable waste for fruits and vegetables from post-intervention to months 6 and 9. These findings are relevant for planning larger trials, as fruits and vegetables are consistently among the most wasted food groups in households [11,12,13,14,27,28]. In the following subsections, we begin by discussing the causes of household fruit and vegetable waste before the intervention and the intervention’s effect on reducing these causes. Also, we discuss the effect of tailored information–enablement interventions on reducing fruit and vegetable waste. Finally, we analyze the follow-up, strengths, limitations and recommendations.

### 4.1. Effect of the Intervention on the Causes of Fruit and Vegetable Waste

Understanding the causes of waste is necessary to design effective interventions. In this context, some authors point out that identifying the causes of waste is a preliminary step to implementing an intervention [28]. Based on the existing literature, we grouped the causes into four intervention pillars [24,48,49] and calculated an index for each cause (purchase, preparation, consumption, storage) for fruit and vegetable categories. Before the intervention, the index indicated that fruit and vegetable purchases were the main causes of waste. This was consistent with previous research indicating that excessive purchasing is a cause of waste in developed countries such as Italy and Australia [16,28]. For developing countries like Chile, data show a similar trend with excessive purchasing and incorrect purchasing as the main causes of waste in Pakistani, Uruguayan, and South African households [15,49,53]. Based on the open-ended comments, we observed that purchase causes may be related to practices such as purchasing during special sales (e.g., 2 × 1) and purchasing products based on novelty or without prior planning (e.g., shopping list). Since fruits and vegetables are highly perishable [2,7], excess or unplanned purchases are expected to be wasted at the household level. Note that some IADL—such as the option not to prepare food, enjoy cooking activities and enough resources to buy fruits and vegetables—may shape purchasing routines and increase the likelihood of overprovisioning (i.e., buying more than needed). Consistent with this, Ananda et al. reported that overprovisioning was associated with high fruit and vegetable waste in Australian households [28].

The index also suggested that preparation, consumption, and storage were less relevant causes of fruit and vegetable waste. We observed good practices such as not serving dressed salads (which allows refrigerating leftovers) and refrigerating or freezing parts of vegetables (which allows their reuse in other meals). Yet we also observed practices related to preparation and storage; for example, some participants indicated a preference for peeled fruits and vegetables (preparation) and for non-refrigerated fruits (storage). Regarding these practices, the consumption of peeled fruits and vegetables not only generates more waste but also eliminates insoluble dietary fiber, an abundant component of the edible peels of fruits and vegetables [7]. On the other hand, refrigeration is a special concern in households, as refrigerated storage of fresh fruits and vegetables extends their shelf life [38].

The previous literature indicates that excessive food preparation and inadequate storage are major causes of waste [15,16,17]. However, preparation, consumption, and storage-related causes appeared less relevant in our study. Differences in the causes of waste highlight the importance of using an index to measure their relevance over time. This approach enables the implementation of tailored interventions targeting the most significant causes of waste. Thus, we focused on recommendations to improve the intervention pillar with the highest index (i.e., the purchase pillar). At the household level, there was a greater opportunity to improve in the pillars, most frequently reported as causes of waste across the different categories of fruits and vegetables. After the intervention, the major differences between the intervention and control groups were observed in fruit and vegetable purchases, in agreement with the tailored intervention’s emphasis. This could be explained by the fact that household heads in the intervention group reported fewer causes of waste after the intervention. It is difficult to compare these findings with previous research because the literature has focused on measuring effectiveness, primarily centering on waste reduction [20,21]. However, measuring the effects on the causes of waste may be a novel approach, as reducing the causes can reflect behavioral changes that may lead to waste reduction over time.

### 4.2. Effect of the Intervention on Avoidable Fruit and Vegetable Waste

Before the intervention, the avoidable waste was 153 (128–180) g/day per person for fruits and 102 (95–124) g/day per person for vegetables. Different methodologies and measurement units are used to quantify fruit and vegetable waste in households. Furthermore, these methodologies have been used in households with diverse consumption patterns in both developed and developing countries. Consequently, comparisons of food waste quantities across studies should be made with caution. For example, data from European households indicate a fruit and vegetable waste of 84 g/day and 90–110 g/day per person, respectively, using a mass balance methodology [54,55]. Research from Western Europe and Greek households indicates fruit and vegetable waste of 63–82, and 202 g/day per person, respectively, using self-reporting methods [12,56]. Furthermore, a study conducted in Pakistani households reported fruit and vegetable waste of 21 g/day per person, measured by direct weighing [49]. Our results seemed to be higher than those reported in the literature using indirect (i.e., mass balance and self-reporting) and direct methodologies (i.e., direct weighting). A previous study showed that differences in the amount of waste between direct and indirect methodologies may stem from indirect methodologies (e.g., self-reporting) underestimating food waste compared to direct methodologies [57]. The difference between the study in Pakistan and the current study using direct weighing could be explained by the national income levels. Pakistan is a low- to middle-income country, while Chile is a high-income country. Higher levels of waste at the consumption level may be expected in high-income countries [2]. However, other factors, including cultural practices, dietary habits, food availability, and household characteristics, may also have contributed to these differences. After the intervention, a greater variability in ∆waste was observed for fruits than for vegetables.

### 4.3. Observational Follow-Up

Several authors recommend repeated post-intervention measurements to characterize trajectories over time and account for day-to-day variability in waste [18,19]. In our study, the follow-up phase was observational because the control group received the intervention after the initial post-intervention assessment (T0). Therefore, the 6- and 9-month measurements (T6 and T9) reflect within-household waste trajectories after intervention exposure in both groups rather than a randomized between-group estimate of medium-term intervention effects. Accordingly, the follow-up does not support causal inference regarding sustained effects, but it does allow monitoring of medium-term patterns in avoidable fruit and vegetable waste. Thus, in future RCTs, the waitlist control group should remain without the intervention until completion of the final follow-up assessment, after which the intervention could be offered.

Note that households showed a decrease in avoidable waste for fruits and vegetables during the observational follow-up. The observed reduction in food waste may be partly attributable to selection bias, whereby households that continued participating in the follow-up assessments were likely more motivated than those who withdrew after the intervention. We also speculate that the decrease in fruit waste during the follow-up may be because adopting behavioral changes and implementing food-handling practices at the household can be gradual over time. In other words, the decrease in waste may not be observed after an initial post-intervention measurement but may be observed in subsequent measurements. In this context, Bonte et al. [21] conducted a systematic review of the effectiveness of interventions to reduce FLW. The authors noted that RCTs ranged widely in duration, from 4 weeks to 32 months (on average, 16 weeks), and found that the longer the time since baseline, the greater the intervention effect. Only one of the studies described by Bonte et al. [21] reported a follow-up within the total study duration (32 months). Thus, they concluded that the time between a baseline and a follow-up measurement can explain differences in effect sizes [21]. However, the effect was smaller for mid- or long-term follow-up. Therefore, previous research on RCTs reinforces the value of follow-up in identifying reductions in fruit and vegetable waste over time.

Finally, although a formal costing or economic evaluation was not conducted, an exploratory cost estimate was performed to inform the feasibility of future studies. The estimate considered fixed costs, such as the development and adaptation of intervention materials, staff training, and reusable equipment (e.g., kitchen scales), as well as variable costs, including personnel time, transportation and household visits, printed materials, and communications. Without considering monetary and non-monetary incentives, the estimated implementation cost for the intervention was CLP 86,000 (approximately USD 95) per household. When the proposed incentives were included, the estimated cost increased approximately 3.7-fold, with incentives accounting for approximately 73% of the total estimated cost. These exploratory estimates indicate that participant incentives would be the main driver of the increase in implementation costs. Therefore, future larger trials should explore lower-cost strategies to support recruitment and retention, including non-monetary incentives or modest incentives that do not substantially increase the cost per household. The incremental benefit of each strategy on recruitment and retention should be evaluated relative to its additional cost. Greater use of digital materials, electronic questionnaires, and optimized household visits may further reduce operational expenses. Because these estimates were not derived from a formal cost-effectiveness analysis, they should be interpreted cautiously.

### 4.4. Strengths, Limitations and Recommendations

This study has several strengths. First, avoidable fruit and vegetable waste was quantified by direct weighing in households rather than relying exclusively on self-reported waste, providing a more objective assessment of the primary outcome. Second, the intervention was tailored according to the causes of waste identified in each household and combined informational content with practical enablement tools, including household-specific purchase lists and recipes. Third, the controlled design allowed an immediate between-group comparison after the intervention, while the repeated household measurements provided preliminary information on waste trajectories after intervention exposure. Therefore, this study contributes evidence from Chile, a high-income country in Latin America, where evidence from household-level food-waste interventions using direct waste measurement remains limited.

Limitations should also be considered when interpreting the findings. Households were recruited through convenience sampling and voluntary participation. The sample was relatively homogeneous and comprised predominantly women with post-secondary education and relatively high household income, and residents of the Metropolitan Region of Chile. Recruitment did not reach the initially planned sample size, and participant dropout reduced the number of households available for the observational follow-up analyses. With 46 households included in the immediate post-intervention comparison, the study had sufficient statistical sensitivity only to detect relatively large between-group differences; therefore, smaller but potentially relevant effects may have gone undetected. Nevertheless, the variability and effect estimates obtained in this pilot study may help inform sample size calculations for future trials. Households that agreed to participate and remained in the study during follow-up may also have been more motivated to reduce food waste than those who withdrew, potentially introducing selection bias. Also, participants and study personnel were not blinded to group allocation because of the nature of the intervention.

The questionnaires were administered face-to-face but were based on participants’ perceptions and may therefore have been affected by social desirability or interviewer-related bias. The fruit and vegetable consumption reported by the household head was also considered representative of household consumption, although it may not accurately reflect the intake of all household members. Furthermore, the follow-up phase was observational. Because the waitlist-control group received the intervention after the immediate post-intervention assessment, no untreated control group was available at 6 or 9 months. Intervention recommendations were reinforced through telephone calls and/or household visits during follow-up. Consequently, the follow-up findings describe changes in waste after intervention exposure and continued reinforcement but do not provide a causal estimate of the sustained effect of the initial 3-week intervention.

Future trials should maintain an untreated control group until completion of the final follow-up assessment, after which the intervention could be offered to control households, thereby preserving the ability to estimate sustained intervention effects. These trials should recruit a larger and more socioeconomically, demographically, and geographically diverse sample, potentially using stratified or cluster sampling to improve representativeness [58]. Recruitment and retention strategies should be prespecified and may include regular contact with participants and carefully evaluated monetary or non-monetary incentives. A shorter, clearly defined follow-up period, with assessments conducted, for example, at 1 and 4 months after the intervention, could reduce loss to follow-up while providing information on the early maintenance of behavioral changes. Therefore, the total study duration would reduce from 49 to 28 weeks, which is expected to improve the feasibility of implementing RCT follow-up. To improve scalability, intervention delivery and data collection should prioritize low-cost resources, including digital educational materials, electronic questionnaires, trained field teams, and a limited number of household visits. The costs and potential retention benefits of participant incentives should be formally evaluated before large-scale implementation. Finally, the variability and effect estimates obtained in this pilot study may inform the sample-size calculation and operational planning of a larger randomized controlled trial.

## 5. Conclusions

In this pilot RCT conducted in 46 households in Santiago, Chile, purchasing-related issues were the most frequently reported drivers of fruit and vegetable waste. At baseline, only 26% of households met the ‘5-a-day’ recommendation, and most participants were independent in purchasing and cooking, suggesting the potential to embed waste-prevention practices into everyday routines. The 3-week tailored informational–enablement intervention reduced the frequency of perceived causes of waste, particularly those related to purchasing. Compared with the control group, the intervention reduced avoidable vegetable waste at the post-intervention time point. At the same time, no immediate between-group difference was observed for fruit waste, consistent with the observed effect sizes (0.78 and 0.25 for vegetable and fruit ΔWaste, respectively). Waste levels continued to decline during the 6- and 9-month follow-up. However, because the control group received the intervention after T0, these follow-up results reflect post-intervention trajectories in an observational phase rather than a randomized estimate of sustained effects. This is consistent with evidence that long-term sustained effects are often not evaluated by follow-up in food-waste intervention trials. Overall, the study provides preliminary evidence of the feasibility of repeated direct-weighing assessment and household-tailored delivery based on the main identified drivers. An exploratory cost estimate indicated an implementation cost of CLP 86,000 (approximately USD 95) per household, excluding participant incentives. Because the inclusion of monetary and non-monetary incentives was estimated to increase the cost approximately 3.7-fold, future larger trials should prospectively assess whether lower-cost or non-monetary incentives improve recruitment and retention sufficiently to justify their additional cost. Other strategies, including trained field teams, electronic questionnaires, digital materials, and optimized household visits, may also help reduce implementation costs.

Future larger trials should include a larger and more socioeconomically, demographically, and geographically diverse sample, maintain an untreated control group throughout follow-up, and incorporate prespecified recruitment and retention strategies. A shorter follow-up schedule and greater use of low-cost digital resources may improve feasibility, although the costs and benefits of participant incentives should be formally evaluated before large-scale implementation.

## Figures and Tables

**Table 1:** Characteristics of household heads.

	All	Control	Intervention
n	46	23	23
Sex			
Women	39 (85%)	21 (91%)	18 (78%)
Men	7 (15%)	2 (9%)	5 (22%)
Age	48 (41–59)	46 (37–54)	54 (41–63)
Country of birth			
Chile	45 (98%)	22 (96%)	23 (100%)
Other	1 (2%)	1 (4%)	0 (0%)
Educational level			
Elementary school	1 (2%)	0 (0%)	1 (4%)
Secondary school	4 (9%)	2 (9%)	2 (9%)
Post-secondary degree	41 (89%)	21 (91%)	20 (87%)
Occupation			
Employed	33 (72%)	18 (78%)	15 (65%)
Full-time homemakers	10 (22%)	5 (22%)	5 (22%)
Retired	3 (6%)	0 (0%)	3 (13%)
Income (CLP)			
<500,000	4 (9%)	0 (0%)	4 (17%)
>500,000	42 (91%)	23 (100%)	19 (83%)
Perception of memory			
Bad	6 (13%)	0 (0%)	6 (26%)
Regular	8 (17%)	2 (9%)	6 (26%)
Good	32 (70%)	21 (91%)	11 (48%)

Continuous variables are expressed as median (25th percentile–75th percentile). Categorical variables are expressed as frequency (%). CLP: Chilean pesos. Data provided by the household head.

**Table 2:** Descriptive data on IADL and fruit and vegetable consumption.

	All	Control	Intervention
Buy independently	43 (94%)	23 (100%)	20 (87%)
Prepare food independently	46 (100%)	23 (100%)	23 (100%)
May choose not to prepare food	37 (86%)	19 (83%)	18 (78%)
Enjoy cooking activities	38 (83%)	20 (87%)	18 (78%)
Enough resources to buy fruits	43 (94%)	23 (100%)	20 (87%)
Enough resources to buy vegetables	42 (91%)	23 (100%)	19 (83%)
Frequency of consumption of fruits (days/week)	7 (4–7)	7 (6–7)	5 (3–7)
Servings of fruits (n/day)	2 (1–2)	2 (1–3)	1 (1–2)
Frequency of consumption of vegetables (days/week)	7 (5–7)	7 (5–7)	6 (5–7)
Servings of vegetables (n/day)	2 (1–3)	2 (1–3)	2 (2–3)
Servings of fruits and vegetables (n/day)	4 (3–5)	4 (3–5)	4 (3–5)
5 a day achievement	12 (26%)	6 (23%)	6 (23%)

IADL: Instrumental Activities of Daily Living. Categorical variables are expressed as frequency of “yes” (%). Continuous variables are expressed as median (25th percentile–75th percentile). Data provided by household heads.

**Table 3:** Index for the causes of waste and avoidable waste pre-intervention.

	Total	Control	Intervention
	Pre	Pre	Pre
Fruits			
Purchase (%)	67 (33–97)	67 (33–83)	56 (33–100)
Preparation (%)	22 (11–33)	22 (11–33)	22 (11–33)
Consumption (%)	33 (27–47)	40 (27–47)	33 (23–50)
Storage (%)	25 (10–42)	25 (8–42)	25 (17–42)
Avoidable fruit waste (g/day per person)	153 (128–180)	153 (129–196)	143 (124–170)
Vegetables			
Purchase	48 (17–67)	57 (26–67)	43 (12–57)
Preparation	19 (10–33)	24 (13–33)	17 (7–29)
Consumption	30 (18–48)	34 (19–49)	26 (19–54)
Storage	18 (7–32)	14 (7–20)	19 (13–36)
Avoidable vegetable waste (g/day per person)	102 (95–124)	102 (94–129)	102 (95–123)

Continuous variables are expressed as median (25th percentile–75th percentile).

## Data Availability

The data presented in this study are available on request from the corresponding author. The data are not publicly available due to privacy restrictions.

## Abbreviations

The following abbreviations are used in this manuscript:

CLP	Chilean pesos
FLW	Food loss and waste
IADL	Instrumental Activities of Daily Living
RCT	Randomized controlled trial
